# A Comparative Evaluation of Medicated Oils Prepared Using Ayurvedic and Modified Processes

**DOI:** 10.4103/0250-474X.59548

**Published:** 2009

**Authors:** P. Lahorkar, K. Ramitha, V. Bansal, D. B. Anantha Narayana

**Affiliations:** Herbal Research Laboratory, Hindustan Unilever Research Centre, 64 Main Road, Whitefield, Bangalore-560 066, India

**Keywords:** Ayurveda, herbalised oil process changes, medicated oils, oil soluble extractives

## Abstract

Medicated oils prepared using process as mentioned in Ayurveda are used for external and internal administrations to treat various disorders. *Taila pak vidhi* provides detailed description of such processes. Medicated oils are prepared by prolonged cooking of sesame oil with pasty mass of herbs and decoction of herbs in presence of large quantity of water. We report preliminary findings of physicochemical and chromatographic profiles of changes brought out by such processes and the role of each component. Changes observed when the processes were altered to deviate from those prescribed in Ayurveda are also reported.

Ayurveda, a holistic health care system prescribes usage of different medicated oils for application on the body, with or with out massage for providing health benefits and to treat specific indications. While most of medicated oils are for external usage, certain types of medicated oils that are processed with milk are administered orally also. Detailed medicated oil processing is described in Ayurvedic textbooks recognized by Drugs and Cosmetic Act[1] and in the Ayurvedic Formulary of India (AFI)[2]. Medicated oils have principally three components namely, *drava* or *qwatha* (a liquid which may be aqueous decoction of one or more herbs, or juice of herbs or milk), *kalka* (a fine paste of the herbs) and *sneha dravya* (a vegetable oil). Normally crude sesame oil (SO) is used as *sneha dravya*, though occasionally castor oil and coconut oil is also used either in parts or in full. As per AFI, unless otherwise given for any specific Ayurvedic oil recipe, the ratio of the three components are, *kalka* one part, *sneha dravya* four parts and *drava* should be 16 parts. The general process is that herbs are ground to get coarse powder (# 40) and mixed with just sufficient quantity of water to get a pasty mass to obtain the *kalka.* The raw or powdered herbs (# 10-30) is moistened with water and boiled with 16 times by volume of water to that of herb quantity and continued boiling to reduce the volume to one forth. The decoction is strained using a muslin cloth to obtain the *qwatha* (also written as *kwatha* some times). SO is taken in a vessel and heated for some time; mixed the pasty mass and the aqueous decoction together. This mixture is boiled on mild fire with stirring to avoid *kalka* to adhere to the vessel and boiling continued till all the water evaporates. Ayurvedic process prescribes to boil either till all the water from the decoction evaporates or the moisture in the pasty mass also evaporates. Well-cooked oil should not have any residual moisture (less than 0.1%). The oil is strained while warm through muslin cloth and allowed to cool. As per AFI, when processing is to be done where aqueous decoction is used as one component, the cooking processes need to be done for many days and up to 5 d in some cases.

A brief survey of number of oils listed in AFI showed as low as two herbs and as high as 73 herbs form the composition of different Ayurvedic oils. A literature survey of relevant publications did not yield reports of studies related to possible changes in the property of sesame oil, extraction of herbal components in to the sesame oil, role of using herbs in both pasty form and decoction form. The literature survey also did not show any studies done to report changes seen if process changes are done. It is well known that vegetable oils show degradation and development of rancidity due to heat and exposure to moisture. In the process described above both these factors are involved intensely for long periods, up to 5 d. The processes are obviously energy intensive involving lot of boiling and cooking, and hence add to cost of processing.

The current preliminary investigation was undertaken to generate data on physico-chemical properties, including chromatographic profiles, stability of sesame oil and extraction of herbal components in to the sesame oil. It is very difficult to perform a study on Ayurvedic medicated oils which has a large number of herbs used in the formulation. Hence in this study three herbs representative of different parts of plant were selected, *neem* leaves (*Azadirachtaa indica*), *Manjista* stems (*Rubia cordifolia*), *Mulethi* rhizome (*Glycyrrhiza glabra*). The oil was prepared using one herb at a time and using *kalka* alone and *kalka* and *qwatha* together, and adding the second and third herb in the same sequence. Several modifications were also done in the process related to proportion of *qwatha*, time of cooking, cooking in opened vessel and closed vessel. The scheme of preparation of the oil is given in (Table 1).

**TABLE 1:** SCHEME OF OIL PREPARATION

Sample code	Composition	Proportions of different ingredients
O1	Plane sesame oil cooked with water	Oil+water (1:4)
O2	SO+*Neem (kalka)*	Oil+*kalka* (4:1)
O3	SO+*Neem (kalka+qwatha)*	Oil+*kalka+qwatha* (4:1:16)
O4	SO+*Neem+Mulethi (kalka)*	Oil+*kalka (Neem+Mulethi)* (4:1)
O5	SO+*Neem+Mulethi (kalka+qwatha)*	Oil+*kalka (Neem+Mulethi)+qwatha (Neem+Mulethi)* (4:1:16)
O6	SO+*Neem+Mulethi+Manjista (kalka)*	Oil+*kalka (Neem+Mulethi+Manjista)* (4:1)
O7	SO+*Neem+Mulethi+Manjista (kalka+qwatha)*	Oil+*kalka(Neem+Mulethi+Manjista)+qwatha (Neem+Mulethi+Manjista)* (4:1:16)
O8	SO+*Neem+Mulethi+Manjista*	Cooked in open vessel with 4 times of decoction as that of oil Oil+*kalka (Neem+Mulethi+Manjista)+qwatha (Neem+Mulethi+Manjista)* (4:1:16)
O9	SO+*Neem+Mulethi+Manjista*	Cooked in open cooker with 4 times of decoction as that of oil Oil+*kalka (Neem+Mulethi+Manjista)+qwatha (Neem+Mulethi+Manjista)* (4:1:16)
O10	SO+*Neem+Mulethi+Manjista*	Decoction is 2 times of oil. Oil+*kalka(Neem+Mulethi+Manjista)+qwatha (Neem+Mulethi+Manjista)* (4:1:8)
O11	SO+*Neem+Mulethi+Manjista*	Decoction is equal to oil. Oil+*kalka(Neem+Mulethi+Manjista)+qwatha (Neem+Mulethi+Manjista)* (4:1:4)
O12	SO+*Neem+Mulethi+Manjista*	Decoction prepared by pressure cooking for 1/2 the time taken by traditional method.
		Oil+*kalka (Neem+Mulethi+Manjista)+qwatha (Neem+Mulethi+Manjista)* (4:1:16)
O13	SO+*Neem+Mulethi+Manjista*	Decoction prepared by pressure cooking for 1/4 the time taken by traditional method Oil+*kalka(Neem+Mulethi+Manjista)+qwatha (Neem+Mulethi+Manjista)* (4:1:16)
O14	SO+*Neem+Mulethi+Manjista*	Decoction prepared by pressure-cooking for 1/6 the time taken by traditional method. Oil+*kalka(Neem+Mulethi+Manjista)+qwatha (Neem+Mulethi+Manjista)* (4:1:16)

The table gives formulation details and method of preparation of oils, O1 to O13. SO stand for sesame oil and the numbers in bracket gives the ratio of oil, herb and water used in formulations. These formulations were used for HPLC and HPTLC analysis.

## MATERIALS AND METHODS

### HPLC specifications:

The HPLC analysis was done in Shimadzu-Class VP system using RP-18 column (Phenomenex, 5 μ) using PDA detector. Carried out an isocratic run with methanol: water (70:30), with flow rate 0.4 ml/min. The total run time was 40 min.

### Preparation of oils:

All the herbs were procured from local traders and authenticated by a qualified botanist in our research center. The oils were prepared at a small Ayurvedic pharmacy in a batch size of 1.5 liter each using stainless steel vessels and cooked on gas heaters under supervision of an ayurvedic physician (*Vaidya*). One batch of oil was also prepared by cooking SO using the volume of water same as that of decoction used in other batches, but with out any herb or pasty mass of herb referred as “aqua treated SO”. No antioxidants or stabilizers were added to any of the batches externally. One batch of oil was prepared in a pressure cooker to reduce the time of cooking and obtain removal of water at a faster rate, while all other cooking was done exactly as per AFI process in open vessels. The finished oils were transferred to clean and dry amber color stopperd bottles and stored at ambient temperature. Samples from these batches were used for all analysis. Crude SO from the same batch was used to prepare all the oils. It was observed that the temperature of the oil during the process of cooking was well below 100°, except in the last stage when most of the water has evaporated, it reaches 105-110° for a short while. In the batch where the cooking was done under pressure the temperature could have gone up to 125°.

### Investigations:

All the oil samples were investigated visually for description, color, and were evaluated for odor and physicochemical properties like saponification and iodine values[3]. In order to evaluate any degradation of the sesame oil these oil samples were evaluated for acid and peroxide values[3]. Further they were analysed by gas chromatography standard techniques by using pure reference standards of fatty acids with a view to determine the fatty acid composition[4] and to map any changes in the same. In order to find the percentage of herbal components that could have got extracted in to SO, a literature search was done to find out any published method for determination of extractive values. However no such method was found. The difficulty in finding the extractive values of herbal components dissolved in the oils is due to non-availability of a solvent, which would specifically and selectively extract out either the oil or the herbal components. Hence we developed a method where aqueous methanol was used to extract the herbal components from the cooked oils processed as above.

### Oil soluble extractive value (OSEV) determination:

As mentioned earlier the main difficulty, which we faced during the study, was to find out an effective method to extract the herbal constituents from oils. After trial and error found out that 90% v/v aqueous methanol is comparatively good. The extraction was done by adding 100 ml of 90% v/v aqueous methanol to 50 ml of medicated oil in a conical flask and stirred for 1 h using a magnetic stirrer. After stirring it was covered with aluminum foil and kept in deep freezer at −20° for 2 d. At this stage the oils were seen to be almost solidified, the top methanol layer was filtered immediately through a Watman No. 40 filter paper. This methanol extract was used for further experiments like extractive value calculation and TLC profiling.

For finding out the extractive value, 25 ml of the methanol extract obtained above, was pipetted out in to a previously weighed round bottom (RB) flask and concentrated to dryness. It was then kept under vacuum for 30 min and then in oven for 30 min at 90°. The RB with dried material was weighed accurately and calculated extractive values as percentages.

### Chromatographic studies:

Keeping in mind the broad chemical composition of herbs used to prepare the medicated oil, the oil samples were separated by HPTLC. Four different solvent systems were employed to generate fingerprint profiles for oils. Chloroform:methanol (9:1) and toluene:ethyl acetate (8:2) solvent systems were used for general fingerprinting. Toluene:ethyl acetate:formic acid (5:4:1) and toluene:ethyl acetate:diethyl amine (7:2:1) systems were used for detection of flavonoids and alkaloids, respectively.

For HPTLC finger printing, 10 ml each of methanolic extract of oils were taken and concentrated to 1 ml. From this concentrated extract 10 μl was applied on a pre-coated silica plate (20×10, silica gel 60, F_254_) using Linomat 5 applicator. Each spot was of 8 mm band length. After application the TLC plate was kept aside for few minutes for complete evaporation of solvent present in the spots. After that the plate was kept in a TLC chamber (20×10 cm chamber, Camag make) pre-equilibrated with mobile phase. Allowed the TLC plate to run till 85% of the height and then taken out and kept for drying.

For flavonoids mobile phase used was toluene:ethyl acetate:formic acid (5:4:1), and after visualizing in UV (at 254 and 366 nm) the plates were derivetised with NP-PEG reagent. For alkaloid-like compounds TLC was developed with toluene:ethyl acetate (8:2) as mobile phase and derivatised using Dragendroff reagent. In addition the oil samples were analysed neat (dissolved in n-hexane) by HPLC. For HPLC 1 ml each of the oil samples (Table 1) was taken and diluted up to 10 ml using n-hexane. Solutions were filtered through a microfilter. From the clear solution 10 μl was injected on HPLC column.

## RESULTS AND DISCUSSION

**Fig. 1:**
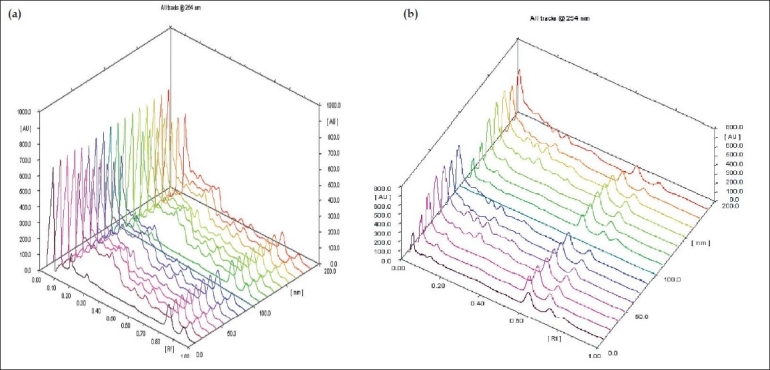
Selected Densitograms of oils The figure shows densitograms of oils obtained after scanning TLC plates at 254 nm. (a) is densitograms of samples O1 to O7 and (b) is densitograms of samples O8-O14.

**Fig. 2:**
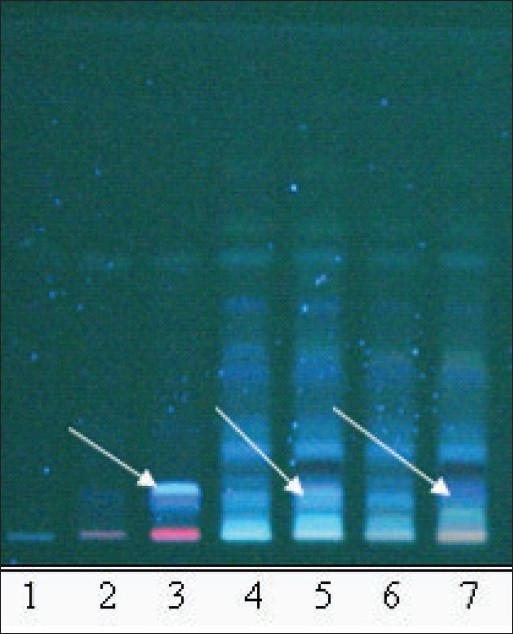
TLC profile of the oil samples (O1-O7). Chromatogram shows difference in intensity/fluorescence of spots according to the formulation changes. Track 1 to 7 is samples O1 to O7 respectively. Spots of higher intensity are pointed with arrows. TLC was done on a silica plate with toluene: ethyl acetate (8:2) as mobile phase. Plate was viewed at 366 nm.

**Fig. 3:**
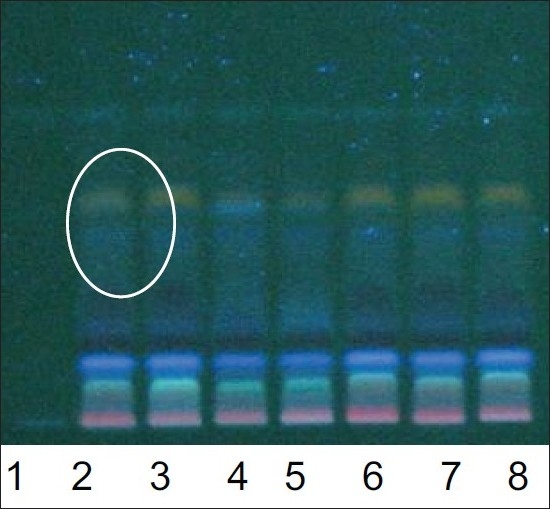
TLC profile of the oil samples (O1, O8 to O14). Chromatogram shows difference in intensity/fluorescence of spots according to the process changes. Track 1 is O1 and track 2 to 8 is samples O8 to O14 respectively. Spots inside the circle are less intense than the others. TLC was done on a silica plate with toluene: ethylacetate (8:2) as mobile phase. Plate was viewed at 366 nm.

**Fig. 4:**
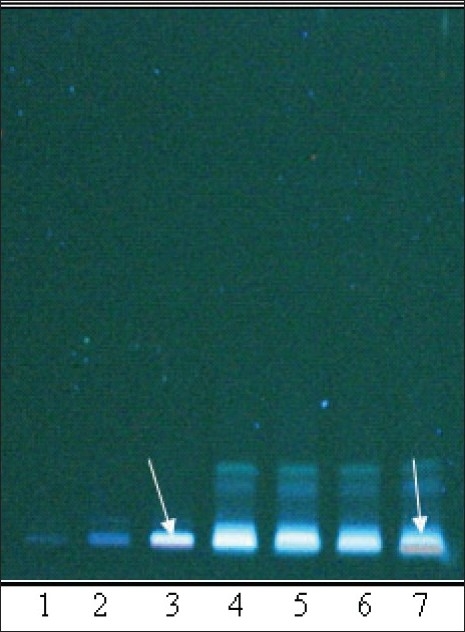
TLC profile of the oil samples (O1 to O7). Chromatogram shows difference in intensity/fluorescence of spots according to the changes in formulation. Track 1 to 7 is samples O1 to O7 respectively. Spots of higher intensity/fluorescence are pointed with arrows. TLC was done on a silica plate with toluene: ethyl acetate: diethyl amine (7:2:1) as mobile phase and plate was viewed at 366 nm.

**Fig. 5:**
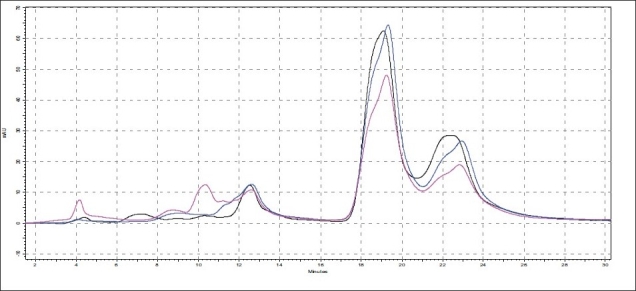
Superimposed HPLC chromatograms of oil samples O1, O2 and O3 Chromatogram shows that area under curve of peak at about 10 min RT is higher in the oil cooked with both *kalka* and *qwatha* in comparison to those only with *kalka*. (
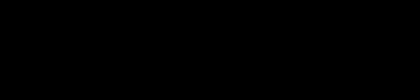
O1, 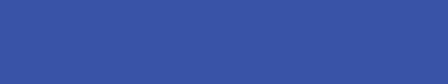
O2, and 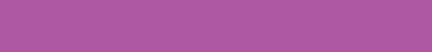
O3).

**Fig. 6:**
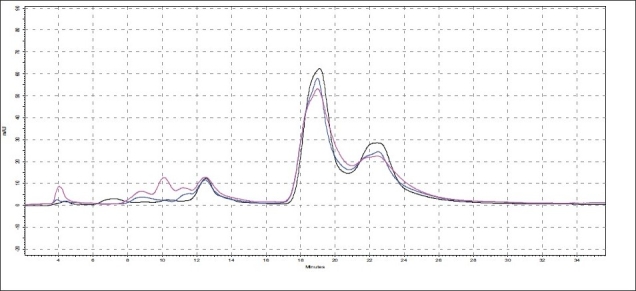
Superimposed HPLC chromatograms of oil samples O1, O4 and O5 Chromatogram shows that area under curve of peak at about 10 min RT is higher in oils cooked with neem and mulethi with qwatha in comparison with kalka alone. (
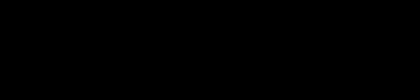
O1, 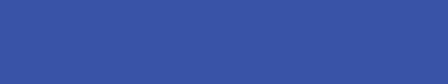
O4, and 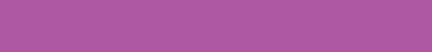
O5).

**Fig. 7:**
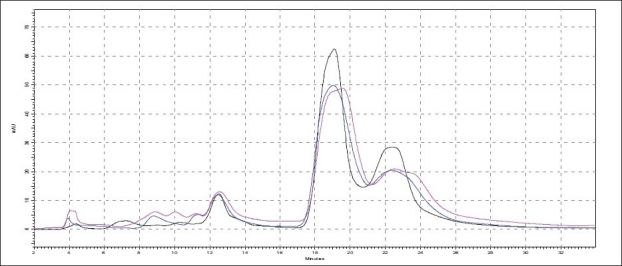
Superimposed HPLC chromatograms of oil samples O1, O6 and O7 Chromatogram shows that the area under curve of peak at about 10 min RT is higher in oils cooked with neem, mulethi and manjishta with qwatha in comparison with kalka alone. (
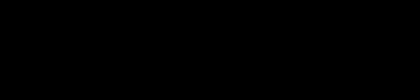
O1, 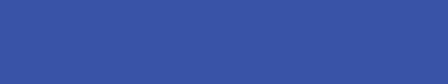
O6, and 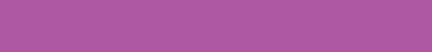
O7).

Results of the visual examination, physicochemical tests and oil soluble extractive values are given in Table 2. Fatty acid compositions of the prepared oils are reported in Table 3. Large number of TLC profiles was obtained during the study, as each separation were viewed under UV 254 nm, UV 366 nm and also viewed after spraying with specific reagents. Since most of the HPTLC profiles showed lot of similarities, all of them are not being provided, but one set of HPTLC chromatograms as their densitometric representations are given in (fig. 1) while (figs. 2 to 4) provide selected TLC profiles of the oils where specific observations were noted. Similarly, HPLC chromatograms of the oils have been presented in (figs. 5 to 7).

**TABLE 2:** RESULTS OF PHYSICO-CHEMICAL TESTS AND OIL SOLUBLE EXTRACTIVE VALUES.

Sample code	Description	Saponification value	Iodine value	Acid value	Peroxide value	Oil soluble Extractive value (%)
SO (crude)[6]	Yellowish with oily note	185-193	103-115	0.5-0.6	10-20	-
Crude SO (used for oil preparation	Yellowish with oily note	189.52	109.95	3.12	11.54	-
O1	Yellowish with oily note	190.00	105.96	3.76	42.76	
O2	Greenish with bitterish note	189.28	107.96	3.89	23.09	0.041
O3	Dark green with burnt, roasted odour	191.03	107.22	4.59	11.25	0.166
O4	Yellowish green with mild oily note	191.76	108.50	4.50	9.02	0.014
O5	Yellowish green with charred note	193.05	107.48	4.52	6.36	0.184
O6	Reddish with slight oily note	190.15	107.20	4.99	6.82	0.158
O7	Reddish brown with burnt oily note.	192.28	107.55	3.71	6.11	0.283
O8	Reddish brown with burnt oily note	192.29	110.18	2.54	11.37	0.393
O9	-Do-	194.52	109.96	2.55	11.13	0.477
O10	-Do-	195.02	110.16	2.71	21.48	0.361
O11	-Do-	194.99	109.15	2.84	21.89	0.389
O12	-Do-	196.39	109.37	2.85	10.86	0.331
O13	-Do-	194.74	109.94	2.71	10.04	0.353
O14	-Do-	196.13	110.63	2.71	11.2	0.366

The table gives the physical parameters like colour and odor and chemical parameters like acid value, iodine value, peroxide value and saponification value of oils O1 to O13, along with the corresponding values for standard sesame oil. The table also gives the oil soluble extractive values.

**TABLE 3:** FATTY ACID COMPOSITION OF THE OILS PREPARED.

Sample code	Palmitic C (16:0)	Palmetolic C (16:1)	Stearic C (18:0)	Oleic C (18:1)	Linolic C (18:2)	Linolenic C (18:3)	Arachidic C (20:0)
Crude SO[7]	7-12	<0.5	3.5-6	35.0-50.0	35.0-50.0	<1	<1
Crude SO (used for oil preparation)	10.61	0.27	5.52	41.61	40.55	0.87	0.55
O1	11.22	0.22	5.24	41.81	41.3	0.22	-
O2	10.89	0.19	5.36	41.85	41.47	0.25	-
O3	10.8	0.2	5.28	41.36	42.04	0.31	-
O4	10.85	0.2	5.43	40.75	42.48	0.3	-
O5	10.2	0.17	4.84	39.64	44.84	0.32	-
O6	11.68	0.25	4.87	39	43.91	0.29	-
O7	10.6	0.24	5.12	41.05	42.57	0.41	-
O8	9.45	0.2	5.14	41.27	42.19	1.43	0.3
O9	10.03	0.21	5.29	41.26	41.23	1.59	0.38
O10	10.04	0.16	5.22	40.88	41.41	1.75	0.53
O11	11.28	0.27	5.47	42.04	39.53	1.01	0.39
O12	10.26	0.21	5.69	42.94	39.61	0.87	0.42
O13	9.42	0.19	5.2	41.02	43.07	1	0
O14	10.3	0.22	5.14	40.7	43.39	0.87	0

The table gives the fatty acid composition of oils O1 to O13 along with corresponding standard sesame oil values.

The different oils gave characteristic color and odour relative to the herb used, and a slight burnt note was seen to higher levels in case of samples were only *kalka* was used. Similarly those cooked under pressure also showed a slight burnt note. The saponification value and iodine value of all the oils prepared irrespective of composition (proportions of *qwatha* as well as absence of *qwatha*, cooking time changes) and change in process were within the normal range for crude SO. SO has about 80% of unsaturation and cooking with oil did not yield any addition to the unsaturated ‘C’ bonds thereby altering the unsaturation levels. It indicates the herbal compounds did not form any linkages with the unsaturated bonds but may be simply dissolving to some extent. There was an increase in acid value in almost all the oils prepared. However peroxide value showed interesting results in those cases where the composition involved cooking SO with water or decoction or heating at higher temperature and pressure gave higher peroxide value. Cooking with *qwatha* showed increase in the amount of herbal components dissolved in oils as compared to cooking with *kalka* alone. It is interesting to note that in spite of cooking the SO with herbs the fatty acid composition of the oil did not show any changes. All the fatty acids tested for was found to be with in the normal range reported for crude SO. Careful viewing of the TLC profile shows some indicative results. The profiles of the TLC separations achieved using various mobile phases and viewed under UV 254 nm as well as sprayed with reagents showed almost similar patterns. The similarities were to a large extent in spite of changes made. However separations achieved using Toluene:ethyl acetate (8:2) as mobile phase viewed under 366 nm shows some changes. Some of the spots appeared to be of a higher intensity when the oil is cooked with *qwatha* in comparison with the oil cooked with *kalka* alone. The same observation was also seen in the TLC profile when separation was done using toluene:ethyl acetate:diethyl amine (7:2:1) viewed under 366 nm. This observation is corroborated in the HPLC separation of oil injected neat. A look at (fig. 5) shows that the area under curve of peak at about 10 min RT is higher in the oil cooked with both *kalka and qwatha* in comparison to those only with *kalka*. Similar observation can be seen in oils cooked with neem and mulethi with *qwatha* as well as neem, mulethi and manjishta with *qwatha* in comparison with *kalka* alone respectively (figs. 6 and 7). Except these observations, the HPLC chromatograms also show great degree of similarities in most of the other parts. It is interesting to note that in spite of cooking in presence of aqueous decoction SO did not show high increase in peroxide value indicating oxidative damage to SO, though hydrolytic damage was seen by increasing in acid value to as much as three to four times of the acceptable range. Authors feel this could be due to presence of antioxidants naturally occurring in SO, mainly sesamol and sesamin[5] and herbal components dissolving in the oil providing additional stabilizing properties. The study also shows that cooking oil with *qwatha* perhaps help get extractive values and components in to the oil. However considering similarities in the components of the oils there appears a potential for changes in proportion of *qwatha* and cooking time consideration, which can be beneficial for reduction in time of cooking and energy consumption. Further studies with one or two traditional oils containing not more than five or six herbs in its composition would provide results of modifications in composition and processing time specific to ayurvedic oil, which can help in cost reduction by way of reducing energy consumption for *qwatha* preparation as well as cooking time. It would be also interesting to evaluate the pharmacological activity of such oils though the proportion of herbal component extracted into SO may be less than one percent and to see the role of such low proportion of herbs providing drug like activity.
